# Supplementary material for: Rational Design of a Portable Chemometric-Assisted Voltammetric Sensor Based on Ion-Imprinted Polymeric Film for Co(II) Determination in Water
Source: Nanomaterials (Basel). 2024 Mar 18;14(6):536. doi: 10.3390/nano14060536 (PMC10976174; doi:10.3390/nano14060536)
Supplement: Supplementary file 1 [file nanomaterials-14-00536-s001.zip › nanomaterials-2876749-supplementary.pdf]

Supplementary Materials

Rational Design of a Portable Chemometric-Assisted Voltammetric Sensor Based on Ion-Imprinted Polymeric Film for Co(II) Determination in Water

Sabrina Di Masi 1,* , Nelson Arturo Manrique Rodriguez 1 , Marco Costa 1 , Giuseppe Egidio De Benedetto 2 and Cosimino Malitesta 1,*

[1] Laboratory of Analytical Chemistry, Department of Biological and Environmental Sciences and Technologies, University of Salento, 73100 Lecce, Italy; nelsonarturo.manriquerodriguez@studenti.unisalento.it (N.A.M.R.); marco.costa@unisalento.it (M.C.)
[2] Laboratory of Analytical and Isotopic Mass Spectrometry, Department of Cultural Heritage, University of Salento, 73100 Lecce, Italy; giuseppe.debenedetto@unisalento.it
* Correspondence: sabrina.dimasi@unisalento.it (S.D.M.); cosimino.malitesta@unisalento.it (C.M.)

Contents

S1. Taguchi experimental design

S2. Electrochemical characterisation

S3. EDX analysis (Energy Dispersive. X-ray Analysis)

S1. Taguchi experimental design

Taguchi orthogonal L9(3^4) array model was selected for optimisation studies during sensor preparation. Each experimental trial (performed randomly as reported in the run order), the Y responses (K_D and IF, respectively), and the S/N ratio for each trial is shown in **Table S1**.

Table S1. Experimental trials from Taguchi orthogonal L9(3^4) array design.

**Experimental run order**	**Sensor name**	**Co(II) concentration, mM**	**2-AP concentration, nM**	**CV scans**	**Time of elution, min**	**Y_1: K_D, nM**	**Y_2: Imprinting factor**	**S/N ratio of Y_1,**	**S/N ratio of Y_2**
2	IIP2	1	0.2	10	10	0.191	2.35	14.3793	7.4214
6	IIP6	1	0.5	20	25	0.060	1.84	24.4370	5.2964
3	IIP3	1	1	30	40	0.188	1.91	14.5168	5.6207
7	IIP7	2	0.2	30	25	0.254	1.24	11.9033	1.8684
9	IIP9	2	0.5	10	40	0.224	1.42	12.9950	3.0458
4	IIP4	2	1	20	10	0.137	3.01	17.2656	9.5713
5	IIP5	3	0.2	20	40	0.308	2.53	10.2290	8.0624
**8**	**IIP8**	**3**	**0.5**	**30**	**10**	**0.029**	**3.42**	**30.7520**	**10.6805**
1	IIP1	3	1	10	25	0.135	2.25	17.3933	7.0437

Each row of the Taguchi model described the condition to be used for sensor preparation. Therefore, all the prepared sensors were assessed towards increased concentration of Co(II) ions dissolved in MES buffer (50 mM, pH 5). Response table for S/N ratio of Y responses is reported in **Table S2**.

Table S2. Response table for S/N ratio of $Y_{1,2}$.

**Level**	**2-AP concentration (mM)**	**Cobalt concentration (mM)**	**CV scans**	**Elution time (min)**
1	8.466	8.934	8.767	12.012
2	9.205	7.582	10.364	7.543
3	10.195	11.350	8.735	8.311
Delta	1.729	3.768	1.629	4.469
Rank	3	2	4	1

Calibration curves are here reported and fitted with Langmuir-Freudlich isotherm function (**Figure S1**).

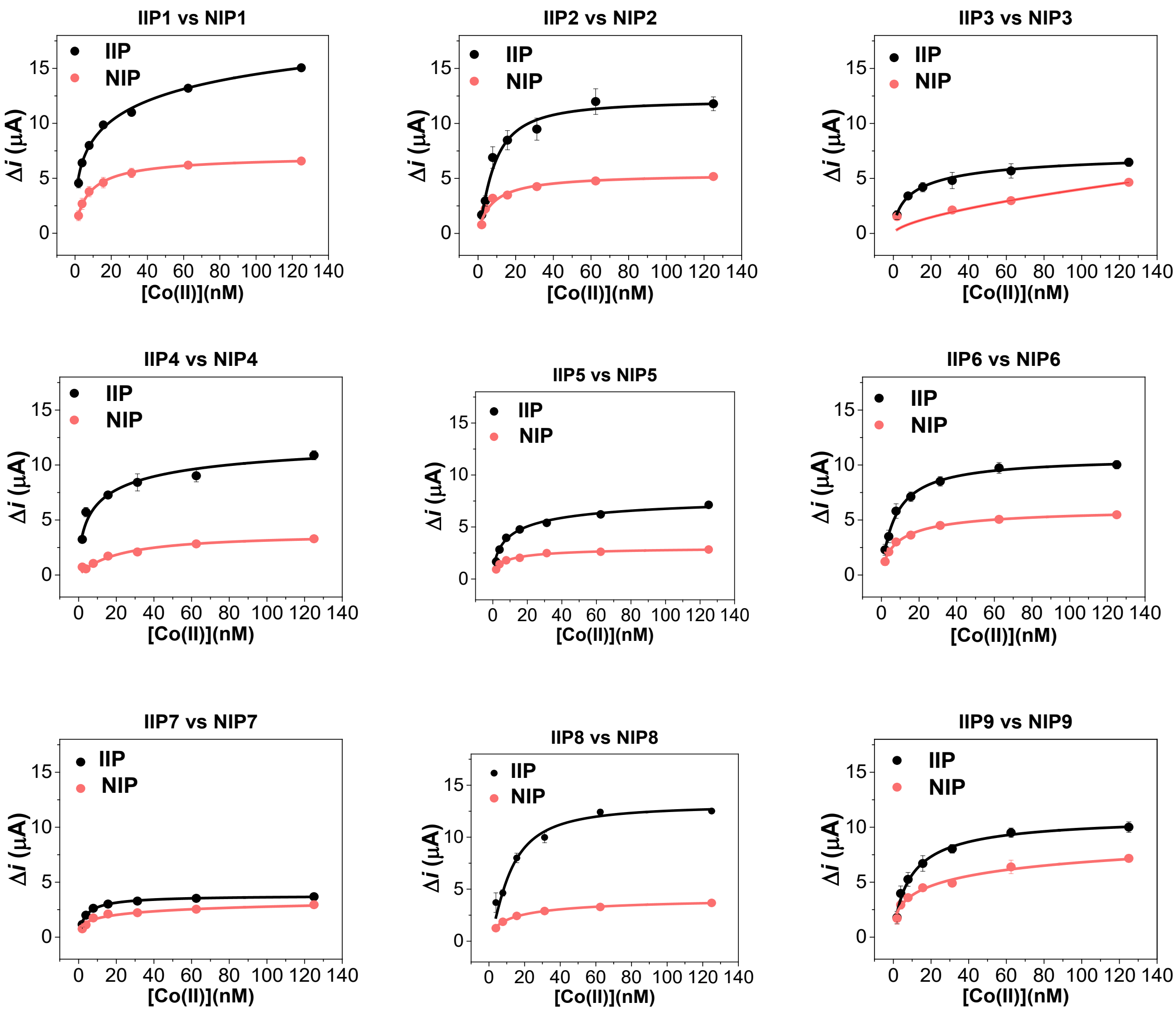

Figure S1. Calibration plots obtained for Co(II)-IIP films and compared to relative NIP films, named IIP1 (vs NIP1), IIP2 (vs NIP2), IIP3 (vs NIP3), IIP4 (vs NIP4), IIP5 (vs NIP5), IIP6 (vs NIP6), IIP7 (vs NIP7), IIP8 (vs NIP8), IIP9 (vs NIP9) developed according to experimental conditions from Taguchi model. Experimental dataset were fitted by Langmuir-Freudlich isotherm function.

From each calibration plots obtained from Co(II)-IIPs films, estimation of kinetics parameters, such as dissociation constant K_D, maximum current per sensor Δi_{max}, and nature of the cavities n have been extracted and reported in **Table S3**.

Table S3. Estimated kinetics parameters from LF isotherm calculated for developed Cd(II)-IIPs films.

Sensor name	K_{LF} ± (nM)	Δi_{max} (μA)	n	R^2
**IIP1**	0.191 ± 0.02	25.13	0.425	0.998
**IIP2**	0.06 ± 0.01	12.10	1.313	0.990
**IIP3**	0.188 ± 0.01	7.68	0.678	0.996
**IIP4**	0.254 ± 0.05	13.21	0.572	0.969
**IIP5**	0.224 ± 0.03	8.25	0.649	0.984
**IIP6**	0.137 ± 0.01	10.72	0.982	0.997
**IIP7**	0.308 ± 0.04	3.81	0.902	0.994
**IIP8**	0.029 ± 0.01	13.10	1.435	0.990
**IIP9**	0.135 ± 0.02	11.02	0.894	0.991
**NIP1**	0.174 ± 0.01	7.10	0.878	0.998
**NIP2**	0.142 ± 0.05	5.43	0.963	0.979
**NIP3**	Null	Null	Null	Null
**NIP4**	0.04 ± 0.004	3.80	1.017	0.995
**NIP5**	0.302 ± 0.04	3.14	0.693	0.990
**NIP6**	0.169 ± 0.01	6.15	0.798	0.998
**NIP7**	0.229 ± 0.09	4.55	0.416	0.951
**NIP8**	0.168 ± 0.01	4.33	0.72	0.997
**NIP9**	0.162 ± 0.05	12.36	0.439	0.978

K_{LF}: Langmuir-Freudlich constant, n: heterogeneity index; Δi_{max}: maximum DPV current change.

S2. Electrochemical characterisation

The electrochemical characterisation by CV (**Figure S2 A,B**) and EIS (**Figure S2 C**) measurements were performed on Co(II)-IIP film and NIP films, respectively.

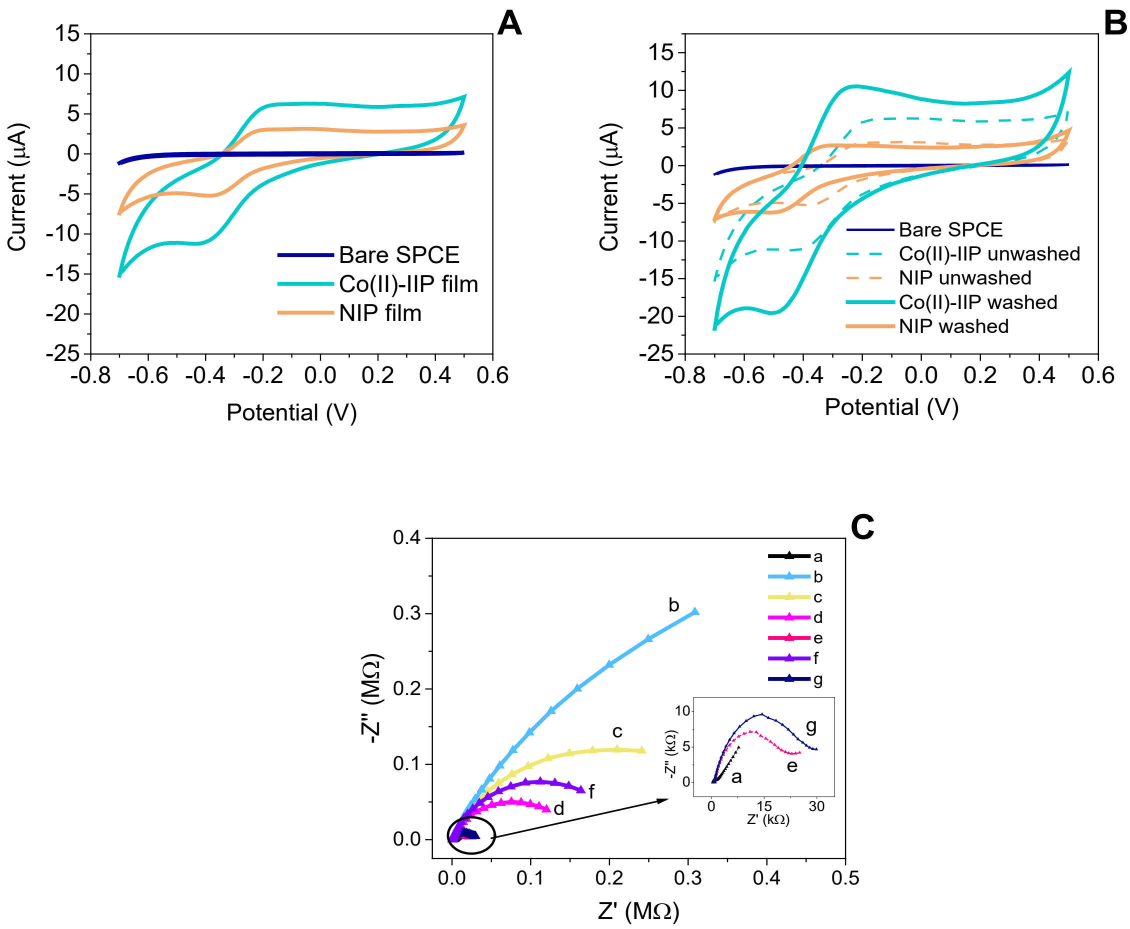

Figure S2. (A) CV measurements for bare electrode, and after preparation of Co(II)-IIP flm and NIP film sensors in MES buffer (50 mM, pH 5). (B) Comparison of CV measurements in MES buffer (50 mM, pH 5) for bare electrode, after Co(II)-IIP film and NIP film preparation and after removing template in 0.3 M H_2SO_4. (C). Nyquist plots in 10 mM $[Fe(CN)_6]^{3-/4-}$ in 0.1 M KCl for different electrodes modification: (a) bare SPCE, (b) after electropolymerisation of Co(II)-IIP film, (c) after electropolymerisation of NIP film, (d) after NIP film wash treatment, (e) after Co(II)-IIP film wash treatment, (f) after NIP film incubation in 31 nM Co(II) solution for 10 min, and (g) after Co(II)-IIP film rebinding for incubation time of 10 min in 31 nM Co(II) ions solution.

The experimental datapoint after each electrode modification were fitted using simplified Randles circuit in the case of NIP films. The Randles circuit elements included the resistance of the solution phase (R), constant phase element (CPE), introduced instead of the double-layer capacitance (C) to take into account the frequency dispersion often related to the electrode roughness, charge-transfer resistance (R_{ct}) and the Warburg diffusion element (W) (used as the additional circuit element in the case of Co(II)-IIP film fitting), accounting for the diffusion of ions from bulk electrolyte to the electrode interface. To investigate the modification of electrode surface at each prepared sensor, triplicate R_{ct} values were collected and compared for interpretation.

S3. EDX spectrum (Energy Dispersive. X-ray Analysis)

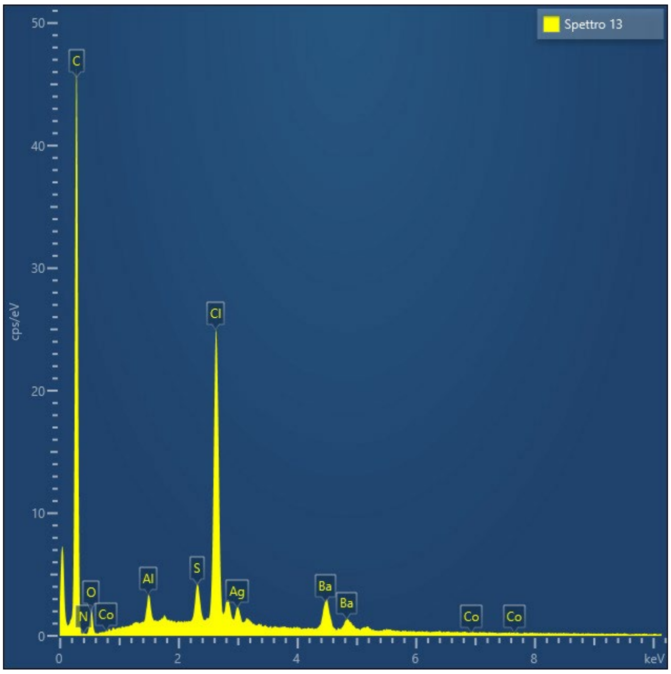

Figure S3. EDX spectrum of washed Co(II)-IIP film sensor.
